# Phosphate Homeostasis in Conditions of Phosphate Proficiency and Limitation in the Wild Type and the *phoP* Mutant of *Streptomyces lividans*

**DOI:** 10.1371/journal.pone.0126221

**Published:** 2015-05-15

**Authors:** Aleksey Smirnov, Catherine Esnault, Magali Prigent, Ian Barry Holland, Marie-Joelle Virolle

**Affiliations:** 1 “Energetic Metabolism of *Streptomyces”*, Institute for Integrative Biology of the Cell (I2BC), CEA, CNRS, Université Paris-Sud, Orsay, France; 2 Energetic Metabolism of *Streptomyces*, Institute for Integrative Biology of the Cell (I2BC), University Paris-Sud, Orsay, France, Sorbonne Universités, UPMC, Univ. Paris 06, UFR927, Sciences de la vie, Paris, France; 3 “Genomic, Structure and Translation”, Institute for Integrative Biology of the Cell (I2BC), University Paris-Sud, Orsay, France; 4 Institute for Integrative Biology of the Cell (I2BC), Université Paris-Sud, Orsay, France; 5 “Energetic Metabolism of *Streptomyces*”, Institute for Integrative Biology of the Cell (I2BC), University Paris-Sud, Orsay, France; Laurentian University, CANADA

## Abstract

Phosphate, as a constituent of the high energy molecules, ATP/GTP and polyphosphate, plays a crucial role in most of the metabolic processes of living organisms. Therefore, the adaptation to low Pi availability is a major challenge for bacteria. In *Streptomyces*, this adaptation is tightly controlled by the two component PhoR/PhoP system. In this study, the free intracellular Pi, ATP, ADP and polyP content of the wild type and the *phoP* mutant strain of *S*. *lividans* TK24 were analyzed at discrete time points throughout growth in Pi replete and limited media. PolyP length and content was shown to be directly related to the Pi content of the growth medium. In Pi repletion, ATP and high molecular weight (HMW) polyP contents were higher in the *phoP* mutant than in the WT strain. This supports the recently proposed repressive effect of PhoP on oxidative phosphorylation. High oxidative phosphorylation activity might also have a direct or indirect positive impact on HMW polyP synthesis. In Pi sufficiency as in Pi limitation, the degradation of these polymers was shown to be clearly delayed in the *phoP* mutant, indicating PhoP dependent expression of the enzymes involved in this degradation. The efficient storage of Pi as polyphosphate and/or its inefficient degradation in Pi in the *phoP* mutant resulted in low levels of free Pi and ATP that are likely to be, at least in part, responsible for the very poor growth of this mutant in Pi limitation. Furthermore, short polyP was shown to be present outside the cell, tightly bound to the mycelium *via* electrostatic interactions involving divalent cations. Less short polyP was found to be associated with the mycelium of the *phoP* mutant than with that of the WT strain, indicating that generation and externalization of these short polyP molecules was directly or indirectly dependent on PhoP.

## Introduction

Phosphate (Pi) is essential for all living organisms. It is required for the synthesis of crucial components of the cell (nucleic acids, phospholipids etc…) and plays important signalling/regulatory roles *via* phospho-transfer reactions. Pi is also central to the energetic metabolism of the cell since it is a constituent of the high energy molecules, ATP, GTP or polyphosphates molecules indispensable for most metabolic processes. Polyphosphates are linear polymers of tens to hundreds of Pi molecules linked by high energy phosphoanhydride bonds [1,2] important for bacterial adaptation to stress and survival in stationary phase [3,4] [5]. When Pi becomes scarce in the environment, in *Streptomyces*, as in other bacteria, the expression of a two-component regulatory system named PhoR/PhoP is triggered [6], [7], [8]. The latter governs the adaptation of cellular metabolism to Pi scarcity. On one hand, in *Streptomyces*, PhoR/PhoP was shown to control positively the expression of specific phosphatases [9] and a high-affinity Pi-specific transport system (PstSCAB) involved in the scavenging and uptake of traces of Pi present in the environment [10]. In other bacteria`, Pi was also shown to be released intracellularly from the polyP stores by exopolyphosphatases belonging to the pho regulon, to adjust to the requirements of cellular metabolism [10,11,12,13,14]. This ability to store and then release Pi protects the cell against abrupt changes in Pi availability in the growth medium. On the other hand, in *Streptomyces*, PhoR/PhoP was shown to negatively control the expression of genes involved in numerous Pi consuming metabolic processes including nitrogen assimilation, nucleotide biosynthesis, oxidative phosphorylation, glycogen catabolism as well as antibiotic biosynthesis and morphological differentiation [6]. PhoP could thus be considered as a master regulator responsible for the adaptation of cellular metabolism to low Pi availability.

In *Streptomyces*, the study of energy/phosphate metabolism is of particular importance since Pi limitation is a crucial trigger for antibiotic biosynthesis [15,16]. Indeed, it was recently demonstrated that an energy deficit (that usually correlates with Pi limitation) activates the degradation of stored lipids, providing precursors for antibiotic biosynthesis, in the *ppk* mutant of *S*. *lividans* [17]. However, little is known on how the intracellular concentrations of free Pi, ATP and polyP vary with the concentration of Pi in the growth medium. In this paper we examined the impact of deleting the gene encoding the response regulator PhoP [7] on the biosynthesis and fate of polyP, as well as on the intracellular content of free Pi, ATP and ADP, of *S*. *lividans* grown in condition of Pi limitation or repletion.

## Material and Methods

### Bacterial strains, media and growth conditions

*S*. *lividans* TK24 WT [18] and *S*. *lividans* TK24 *phoP*:: *aac* strain [19] were used in this study. 10^6^ viable spores of the *S*. *lividans* strains were spread on the surface of a plate (9 cm diameter) of solid medium R2YE covered by a cellophane disk (Cannings Packaging Limited, Bristol, United Kingdom). The medium was supplemented or not with K_2_HPO_4_ [20]. R2YE not supplemented with K_2_HPO_4_ contained 1 mM free Pi (Pi limitation condition) and that supplemented with K_2_HPO_4_ contains 5 mM Pi (Pi replete condition), as determined with a PiBlue phosphate assay kit (Gentaur, France).

### Determination of cell growth and Pi concentration in the growth medium

Growth of the strains was estimated at intervals by dry biomass quantification of the mycelium scraped from cellophane disks of 4 replicates. To quantify the phosphate taken up by the two strains, the cellophane was lifted at different time points during growth, and three sterile agar cylinders of the growth medium were taken using an inverted Pasteur pipette. These were incubated at 4°C for at least 24 h in 1 ml of distilled water in order to allow the diffusion of Pi from medium to water. The concentration of phosphate was then determined using the PiBlue phosphate assay kit.

### Extraction of total polyP

Two different methods, the Kulaev method [21] that yielded three fractions of polyP of different sizes and the Kornberg method [22] (with some modifications) were used to extract polyP from the WT and the *phoP* mutant strains of *S*. *lividans* TK24 at different time points throughout growth. To do so, half of the wet biomass obtained from one or several pooled plates was used to estimate growth (dry cell weight/DCW) and the other half was used to extract polyP. All assays were performed in triplicate in order to calculate standard deviation.

To prepare polyP with the Kulaev method, 0.25 g of the harvested wet biomass was incubated in 5 ml of 0.5 N HClO_4_ at 4°C for 30 min with continuous stirring, then centrifuged at 12,000 g for 15 min at 4°C. Supernatant 1 contained the acid-soluble polyphosphate fraction (Small Molecular Weight polyP, SMW, approximately 10 residues long) as well as free Pi and nucleoside phosphate. The nucleoside phosphate was removed by absorption on activated charcoal Norit A from VWR. Pellet 1 was extracted with 5ml of 0.05N at 4°C for 30 min and centrifuged in the same conditions as above yielding supernatant 2 and pellet 2. Supernatant 2 contained the alkali-soluble polyphosphate (Medium Molecular Weight polyP, MMW, 20 to 100 hundred residues long). The latter were precipitated by addition of barium acetate (pH 8.2) at a final concentration of 0.1 M, at 4°C, for 24 h after adjusting the pH of supernatant 2 to 8.2 with 0.1 N HCl. The suspension was centrifuged at 5,000 g for 30 min. The precipitate was dissolved in 1ml of buffer (50 mM Tris-HCl at pH 8.0, 6 mM MgCl_2_) and solubilized using Dowex AG-50W (sodium form). Pellet 2 was treated with 5 ml of 0.5 N HClO_4_ for 30 min at 100°C. This hot perchlorate extract contains acid- and alkali-insoluble polyphosphates, thought to be High Molecular Weight polyP (HMW, several hundred residues long).

To prepare polyP by the Kornberg method [22], 0.25 g of the harvested wet biomass was suspended in 750 μl of lysis buffer STE (10 mM Tris-HCl at pH 8.0, 1 mM EDTA, 6 mM MgCl_2_ and 250 mM sucrose) and broken by sonication. The lysate was incubated with proteinase K, 750 μg.ml^-1^, at 37°C for 2 h,then extracted with phenol/chloroform (1:1, w/v equilibrated with Tris-HCl, pH 7.5). The phases were separated by centrifugation at 14,000 g for 10 min. The aqueous phase was transferred to another tube and the phenol layer was back-extracted twice with 50 mM Tris-HCl, pH 7.5, 10 mM EDTA. The pooled aqueous phase was extracted with chloroform. PolyP was precipitated with barium acetate at a final concentration of 0.1 M at pH 4.5 for 24 h at 4°C. The precipitate was collected by centrifugation at 14,000 g for 30 min. The precipitate was dissolved in 1 ml of buffer (50 mM Tris-HCl at pH 8.0, 6 mM MgCl_2_) and solubilized using Dowex AG-50W (sodium form).

The fractions obtained by both methods were incubated with DNase I and RNase A, each at 350 μg.ml^-1^, in 5 mM MgCl_2_ for 3 h at 37°C, then incubated with proteinase K, 750 μg.ml^-1^, at 37°C for 2 h. The samples were subsequently extracted with phenol/chloroform (1:1, w/v equilibrated with Tris-HCl, pH 7.5) to remove proteins. The phases were separated by centrifugation at 14,000 g for 10 min. The aqueous phases (containing polyP) were extracted with chloroform, centrifuged in the same conditions, collected and stored at−20°C.

The amount of Pi and polyP present in the different fractions was determined by the estimation of the difference in the Pi content of the samples before and after the complete hydrolysis of the polyP, following incubation of the samples with 2 M HCl at 100°C for 10 min and using the PiBlue phosphate assay kit.

### Electrophoretic analysis of polyP

Urea-polyacrylamide gels were prepared by mixing 10.51 g of urea, 5 ml of acrylamide solution (40% acrylamide:bisacrylamide, 19:1), and 12 ml of 0.9 M Tris borate (pH 8.3) and 27 mM EDTA, to a final volume of 25 ml. Ammonium persulfate 50 μl (10%) and TEMED (20 μl) were added and the gel was poured (14 x 23 x 1.5 mm). PolyP samples were mixed with 0.25 volume of 5X sample buffer (50% sucrose, 0.125% bromophenol blue, and 450 mM Tris borate at pH 8.3, 13.5 mM EDTA) and loaded on the gel. Electrophoresis was run at 200 V until the dye was 9 to 11 cm from the top of the gel. Gels were stained with 0.05% toluidine blue in 25% methanol and 5% glycerol for 20 min followed by de-staining in the same solvent without toluidine blue. For determination of chain lengths, polyP markers (Sigma) of various sizes were run together with the samples and visualized by toluidine blue staining.

### ADP and ATP extraction and assay

In order to quantify the intracellular concentration of ATP and ADP, 50 mg of mycelium was collected in screw-cap 2 ml tubes (Sarstedt), mixed with 1 ml of 5% cold perchloric acid, frozen in liquid nitrogen and stored at−70°C. Glass beads (0.16 g, 0.5 mm diameter) were added to each tube and the mycelium was homogenized and broken by vigorous shaking (FastPrep). The samples were kept at 4°C, centrifuged at 16,000 g for 10 min, the potassium perchlorate precipitate pellet was discarded and the supernatants were neutralized with KOH 34% and 3.4% and finally with phosphate potassium buffer (1 M at pH 7.5). The ATP and ADP concentrations were determined by the luciferin-luciferase method using an ATP luminescence kit (CSLII, Roche) and a one-sample GLOMAX luminometer (model 2031–002, Turner BioSystems, Inc). The background was measured by mixing 35 μl of 330 mM Tris acetate buffer (pH 7.75), 4 mM EDTA, 21 mM K_2_SO_4_ with 50 μl of luciferin-luciferase mix. The sample (10 μl) was added and after measuring luminescence, 10 μl of an internal ATP standard of *ad hoc* concentration (depending on the [ATP] in the sample) was added and luminescence measured again. [ATP] was determined directly according to a standard curve (0.1 to 10 μM) made in the same conditions. All assays were performed in triplicate in order to calculate standard deviation.

[ADP] was measured after enzymatic conversion to ATP with pyruvate kinase and phosphoenol pyruvate. The background was measured by mixing 35 μl of 330 mM Tris acetate buffer (pH 7.75), 4 mM EDTA, 21 mM K_2_SO_4_, 17 mM phosphoenol pyruvate and 0.57 mg.ml^-1^ pyruvate kinase (Roche) with 50 μl of luciferin-luciferase mix. The sample (10 μl) was added and after measuring luminescence, 10 μl of an internal ADP standard of *ad hoc* concentration (depending on the [ADP] in the sample) was added and luminescence measured again. [ADP + ATP] was determined according to an ADP (2 to 30 μM) standard curve made in the same conditions. [ADP] was calculated by subtracting [ATP] from [ADP + ATP]. Results were expressed as nanomoles of nucleotides per mg of Dry Cell Weight (DCW). All assays were performed in triplicate in order to calculate standard deviation.

### Fluorescence microscopy observations of mycelial fragments stained with DAPI

The microscope slides were treated with 0.1% poly-L-lysine for 5 min at room temperature, then rinsed 3 times with sterile water. Mycelial fragments were smashed and spread on the slide and kept 5 min for fixation. A drop of 50 μg/ml DAPI in 25% glycerol was then added to stain the biomass. The stained biomass was then covered by a 12-mm-diameter round glass coverslip and observed on a DM IRE2 microscope (Leica). Images were captured by a CoolSNAP_HQ2_ CCD camera (Photometrics). MetaMorph software (Universal Imaging Corp.) was used to acquire a z-series, de-convolute the z-series and treat the images. DNA has a fluorescence emission maximum at 456 nm and was detected using the DAPI filter A4 from Leica. PolyP has a fluorescence emission to an higher wavelength than DNA, with a maximum of 525 nm [23] and was detected using the GFP filter (3035B) from Semrock.

### Determination of extracellular level of polyP

The collected wet biomass (0.2 g) was incubated for 10 min with constant stirring in 2 ml of distilled water or in 5 mM EDTA. The supernatant was separated by centrifugation at 14,000 g for 10 min. The total polyP content of the samples was calculated by subtracting the content of orthophosphate before and after hydrolysis with 2 M HCl at 100°C for 10 min as described above. All assays were performed in triplicates in order to calculate standard deviation.

## Results and Discussion

### Internal free Pi concentration is lower in the *phoP* mutant than in the WT strain

**Fig 1:**
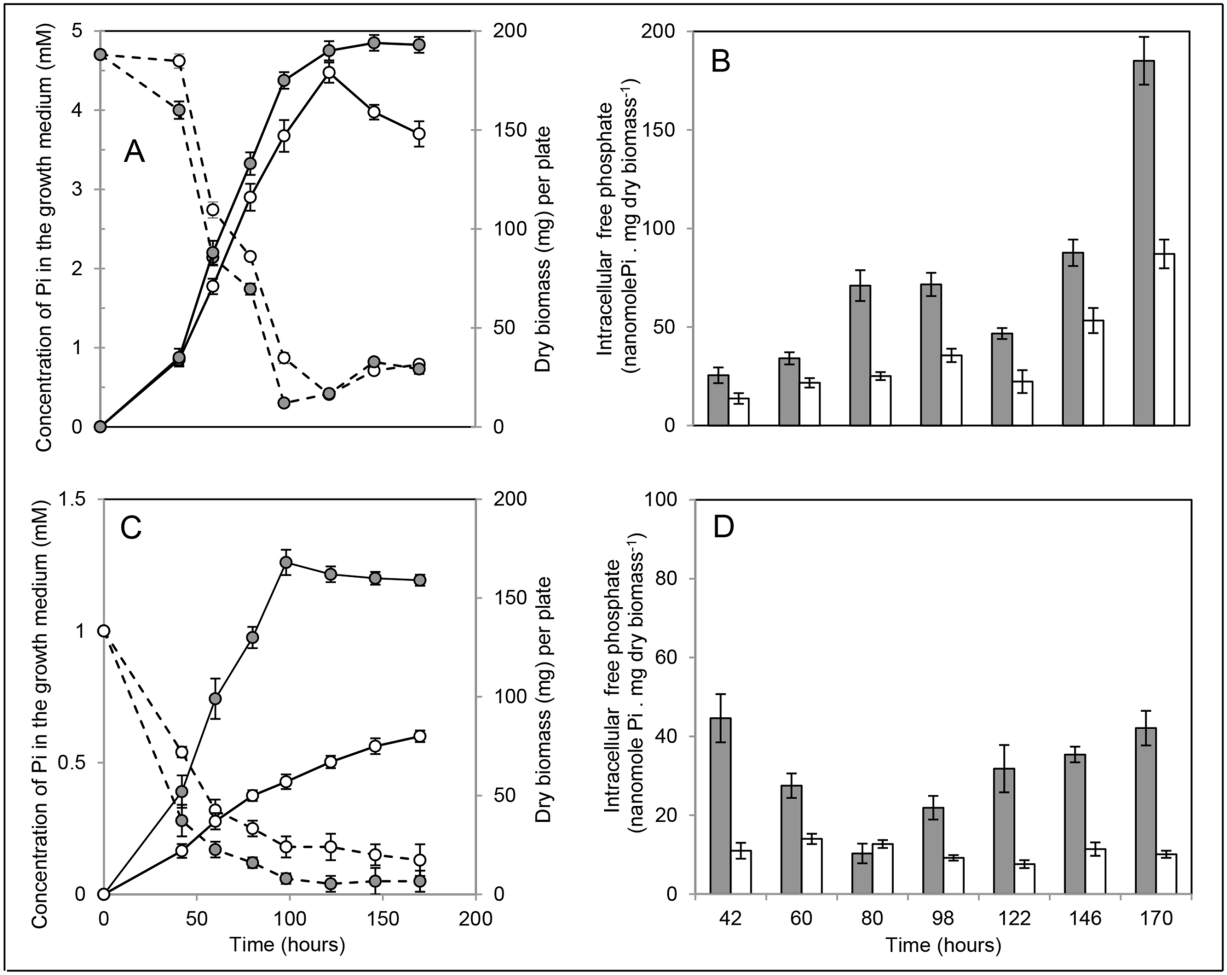
Growth and concentration of free Pi in the medium and inside the cells throughout cultivation. Growth (continuous lines, dry biomass per plate) and concentration of Pi in the growth medium (dotted lines) of the WT (grey circles) and the *phoP* mutant (white circles) of *S*. *lividans* TK24 grown on a Pi replete (A) or Pi limited (C) R2YE solid medium. Intracellular free Pi concentration of the WT (grey histograms) and the *phoP* mutant (white histograms) grown on a Pi replete (B) or Pi limited (D) R2YE solid medium. The error bars represent the standard deviation of three independent experiments.

The WT strain of *S*. *lividans* TK24 and its *phoP* mutant were grown on the surface of a cellophane disk laid down on the R2YE solid medium either limited (1 mM Pi) or replete (5 mM Pi) in Pi. Growth curves, kinetics of Pi uptake and intracellular free Pi concentration are shown in Fig 1A–1D. In conditions of Pi proficiency (Fig 1A), the growth rate of the WT and *phoP* mutant were similar. Both strains reached stationary phase after 122 h of cultivation. However, at stationary phase whereas the biomass of the WT strain remained stable, that of the *phoP* mutant decreased, suggesting cell lysis. The rate of Pi uptake (as measured by disappearance of Pi from the medium) was similar in both strains (Fig 1A). Interestingly, a slight increase of free Pi in the growth medium was observed for both strains, after 122 h of incubation (Fig 1A). A steady increase in the content of intracellular free Pi was observed in both strains, reaching its maximum at late time points when Pi of the growth medium was almost exhausted (Fig 1B). However, this increase was not as pronounced in the *phoP* mutant and the overall intracellular concentration of free Pi of the mutant was lower than that of the WT strain throughout growth, despite similar uptake rates (Fig 1B). This suggested a less efficient release of Pi from the polyP stores in the *phoP* mutant compared to WT.

In conditions of Pi limitation (Fig 1C), the growth profile and the growth yield of the WT and the *phoP* mutant strains were drastically different. The WT strain showed a classical phase of active growth (synthesis of 1.7 mg dry weight.h^-1^) followed by a stationary phase, whereas growth of the *phoP* mutant was slow (synthesis of 0.65 mg dry weight.h^-1^) and relatively linear up to 170 h. As expected, the rate of Pi uptake was slightly slower in the *phoP* mutant than in the WT strain [10]. However, the difference in Pi uptake rate cannot account for the huge difference in biomass yield of the two strains. Results shown in Fig 1D indicated a lower intracellular free Pi content in both strains in these Pi limiting compared to Pi replete conditions. A steady decrease (coincident with active growth from 42 h to 80 h) followed by a steady increase (coincident with stationary phase from 98 h to 170 h) in the content of intracellular free Pi, was observed in the WT strain, reaching its maximum at late time points when Pi of the growth medium was almost exhausted. In contrast, the intracellular content of free Pi in the *phoP* mutant was constant and low (10 nanomole Pi.mg^-1^ biomass) throughout the growth phase. The high level of intracellular free Pi, in the WT strain, observed in both Pi conditions when Pi of the growth medium was almost exhausted, suggested the existence of an early process of storing Pi as polyP (the main phosphate storage polymer) and subsequent mobilization of the Pi stored in these polymers. At stationary phase, the released Pi will accumulate since it is not consumed to support growth. The fact that the overall content of free intracellular Pi was always lower in the *phoP* mutant than in the WT strain throughout the growth phase, in both Pi conditions, suggested the existence of a less efficient degradation of polyP to Pi in the *phoP* mutant, as could be expected. However, we cannot totally exclude that a more efficient storage of Pi as polyP occurs in the mutant. In order to test these two hypotheses, the evolution of intracellular polyP, was assessed throughout growth of the WT and *phoP* strains, on Pi limited and replete R2YE solid medium.

### PolyP degradation is clearly delayed in the *phoP* mutant

In order to assess the evolution of the intracellular polyP content throughout the growth phase in Pi limitation or repletion, polyP size fractionation and quantification was carried out with the Kulaev and/or the Kornberg methods.

**Fig 2:**
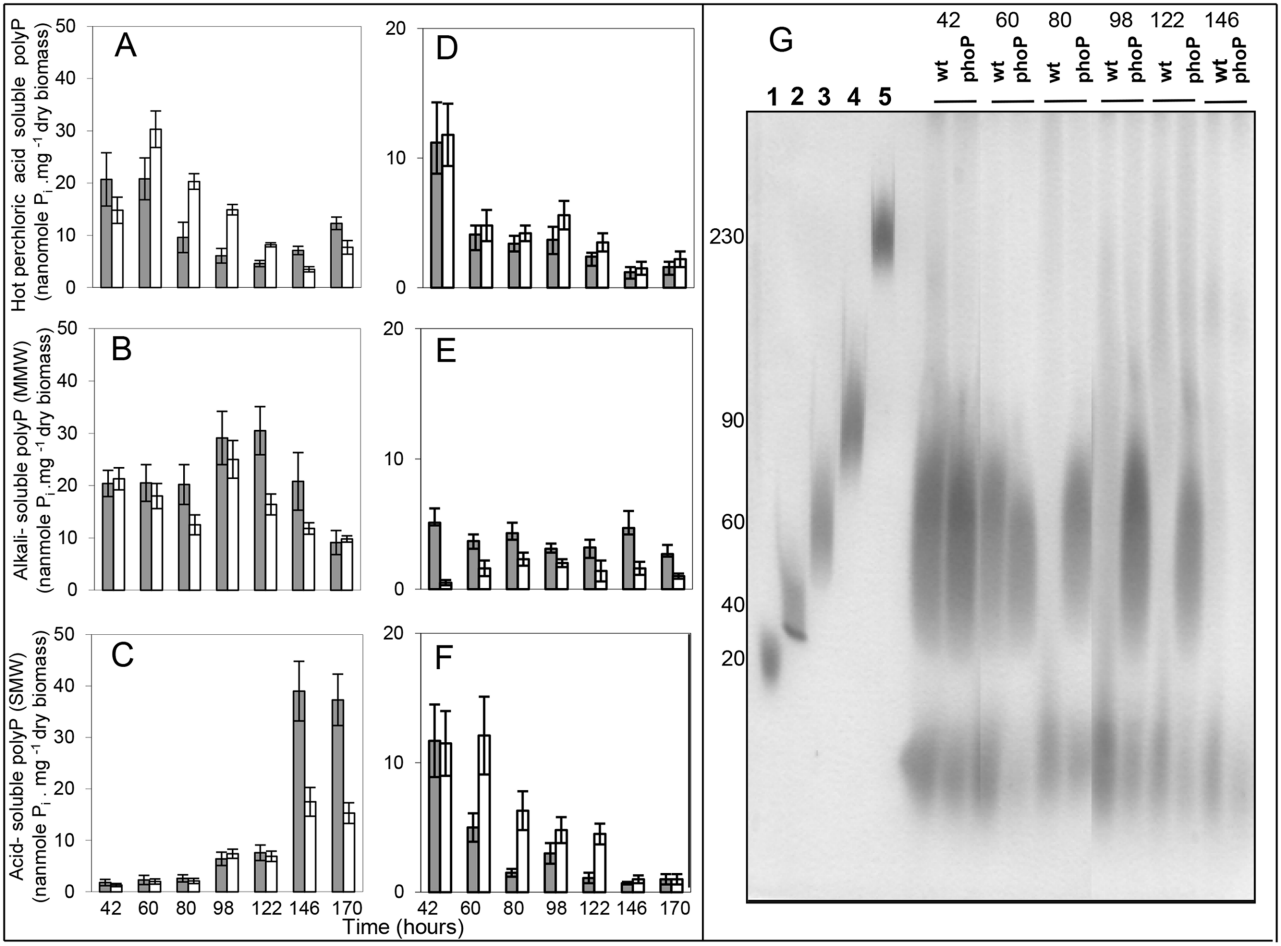
Estimation of the Pi content of polyP fractions extracted with the Kulaev and Kornberg methods. PolyP was first extracted with the Kulaev method from the WT (grey histograms) and the *phoP* mutant (white histograms) of *S*. *lividans* TK24 grown on a Pi replete (A, B, C) or Pi limited (D, E, F, G) solid R2YE medium, at different time points throughout growth. This extraction yielded three fractions: (A, D) hot perchlorate fraction polyP (HMW polyP); (B, E) alkali-soluble fraction polyP (MMW polyP); (C, F) acid-soluble fraction polyP (SMW polyP). PolyP was also extracted with the Kornberg method from the WT and *phoP* mutant, at different time points on Pi limited R2YE medium. PolyP was run on urea-polyacrylamide gel (G). Lanes 1, 2, 3, 4, 5 contain size standard polyP, approximate length 20, 40, 60, 90 and 230 residues. The other lanes contain polyP preparations of the WT and *phoP* mutant. Note that the HMW polyP is not able to enter the gel and is therefore not detected. Only MMW and SMW polyP are visible.

In conditions of Pi proficiency, the level of HMW polyP was high at early times and decreased steadily in both strains (Fig 2A). The content of HMW polyP was higher in the *phoP* mutant than in the WT strain throughout growth (Fig 2A). In contrast, the level of MMW, SMW polyP and free Pi was lower in the *phoP* mutant at late time points (Figs 2B, 2C and 1B). The decrease in the content of MMW polyP at late time points in both strains (Fig 2B) correlated with an increase in the content of SMW (Fig 2C) and free Pi (Fig 1B) at 146 h and 170 h. This increase was more abrupt in the WT strain than in the mutant and at 146 h and 170 h the WT contained at least two fold more SMW polyP than the mutant (Fig 2C). Altogether these data suggested that less active degradation of HMW polyP into shorter forms and Pi occurred in the *phoP* mutant compared to the WT strain.

In conditions of Pi limitation, polyP size fractionation and quantification by the Kulaev method (Fig 2D, 2E and 2F) and preparation and visualization by the Kornberg method (Fig 2G), respectively, showed that the overall polyP content was, as expected, lower in Pi limitation than in Pi sufficiency. HMW polyP was present in comparable low levels in the WT and the mutant strains (Fig 2D), whereas MMW polyP seemed slightly less abundant in the *phoP* mutant than in the WT (Fig 2E). In contrast, SMW polyP was more abundant in the mutant than in the WT strain throughout most of the growth phase (Fig 2F). Visualization of MMW and SMW polyP prepared by the Kornberg method (Fig 2G) confirmed that the latter was hardly visible after 80 h in the WT whereas, it remained detectable up to 122 h in the mutant. SMW polyP (Fig 2F and 2G) and intracellular free Pi (Fig 1D) were consistently more and less abundant, respectively, in the *phoP* mutant than in the WT strain. Altogether these data suggested that less efficient degradation of SMW polyP into Pi occurred in the *phoP* mutant compared to the WT strain, as was previously noted in Pi proficiency.

The low concentration of intracellular free Pi in the mutant in conditions of Pi limitation was anticipated to have a negative impact on ATP synthesis. In order to verify this, the intracellular ATP and ADP concentrations were assayed in both strains throughout growth in conditions of Pi repletion and limitation, as described below.

### Assay of ATP concentration in the WT and *phoP* mutant strains confirmed the putative repressive effect of PhoP on oxidative phosphorylation

**Fig 3:**
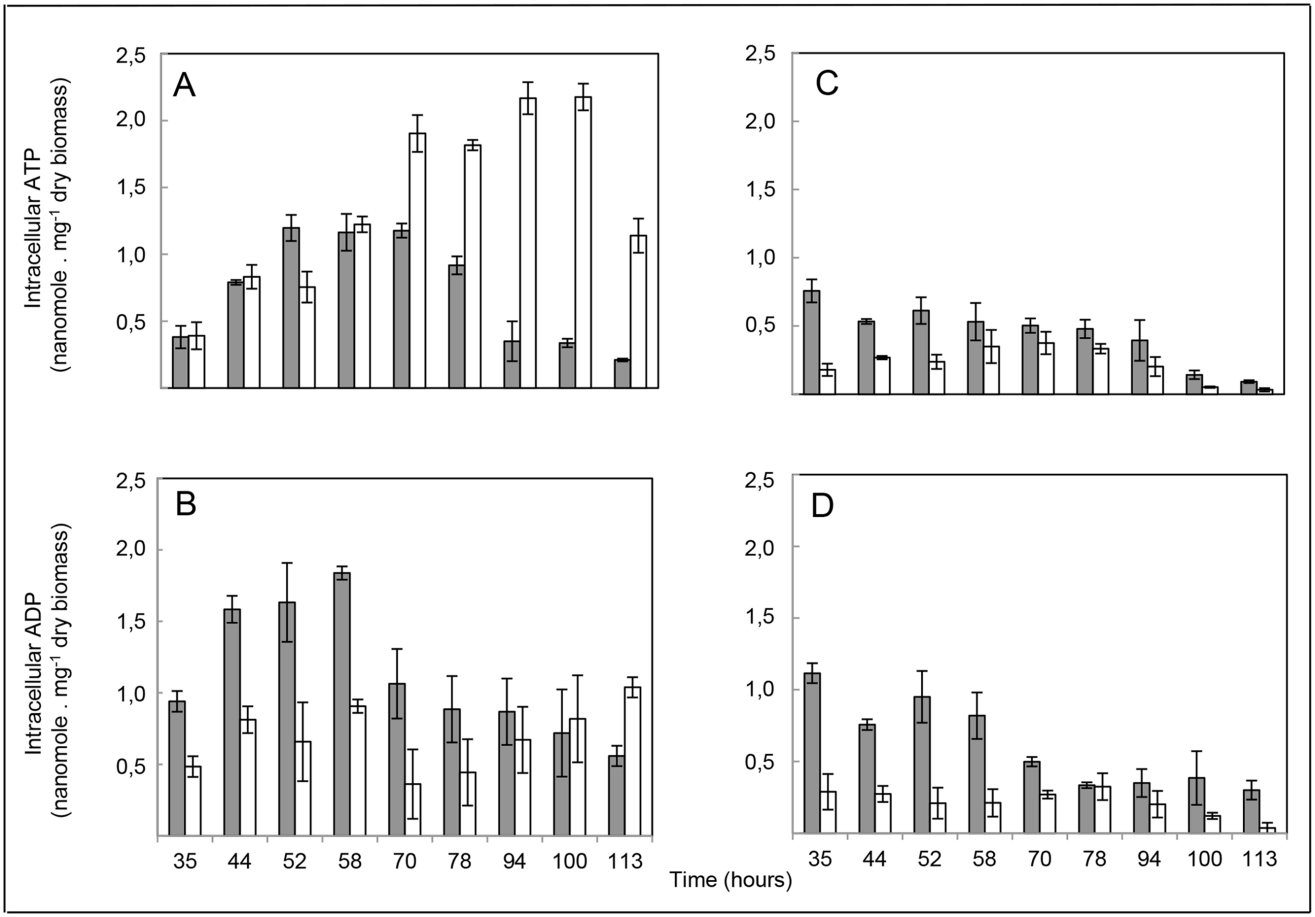
Quantification of intracellular ATP and ADP levels. Intracellular ATP (A, C) and ADP (B, D) levels were quantified in the WT (grey histograms) and the *phoP* mutant (white histograms) grown on a Pi proficient (A, B) or Pi limited (C, D) medium. The error bars the represent the standard deviation of at least three independent experiments.

ATP and ADP were assayed at discrete points during the growth phase of the two strains grown on the R2YE solid medium either limited (1 mM Pi) or replete (5 mM Pi) in Pi. As anticipated, ATP and ADP concentrations were reproducibly lower in both strains in Pi limitation than in Pi proficiency (Fig 3A–3D).

In conditions of Pi repletion (Fig 3A and 3B), the ATP content of both strains was approximately the same up to the 58 h of incubation. After this point, the ATP content of the WT decreased whereas, unexpectedly, that of the *phoP* mutant kept increasing up to 100 h and decreased thereafter. In the WT strain, the ADP and ATP concentration followed a similar temporal pattern. The ATP/ADP ratio was > or = to 1, up to 78 h and then fell below 0.5 suggesting a defect in ATP regeneration from ADP in that strain. In the *phoP* mutant, the ADP and ATP concentration followed a very different temporal pattern. The ATP/ADP ratio was > or = to 1, up to 58 h, then became > or = to 4. This unexpected observation indicated that an efficient ADP into ATP regeneration process was taking place in the *phoP* mutant after 58 h and up to 100 h of cultivation. This is consistent with the recent publication of Allenby *et al*. [6] demonstrating that PhoP represses the expression of genes linked to the respiratory chain and thus oxidative phosphorylation. The relief of this repression in the *phoP* mutant would account for the unexpectedly high level of ATP observed in this strain, in these conditions. In conditions of Pi limitation (Fig 3C and 3D), the overall ATP content of the WT was approximately 2 fold higher than that of the mutant strain up to 52 h but reached similar levels afterwards (Fig 3C). In the WT strain the ATP/ADP ratio was close to 0.5, up to 58 h and close to 1, subsequently. In the *phoP* mutant, the ATP/ADP ratio was close to 1 throughout growth. Interestingly, the ADP concentration was 3 to 4 fold lower in the *phoP* mutant than in the WT strain up to 70 h of incubation, suggesting either a reduced synthesis of this nucleoside or a more efficient oxidative phosphorylation (ADP to ATP regeneration) in the *phoP* mutant, in Pi limitation, as in Pi repletion (Fig 3D).

### Short polyP is found associated with the surface of *Streptomyces* mycelium

**Fig 4:**
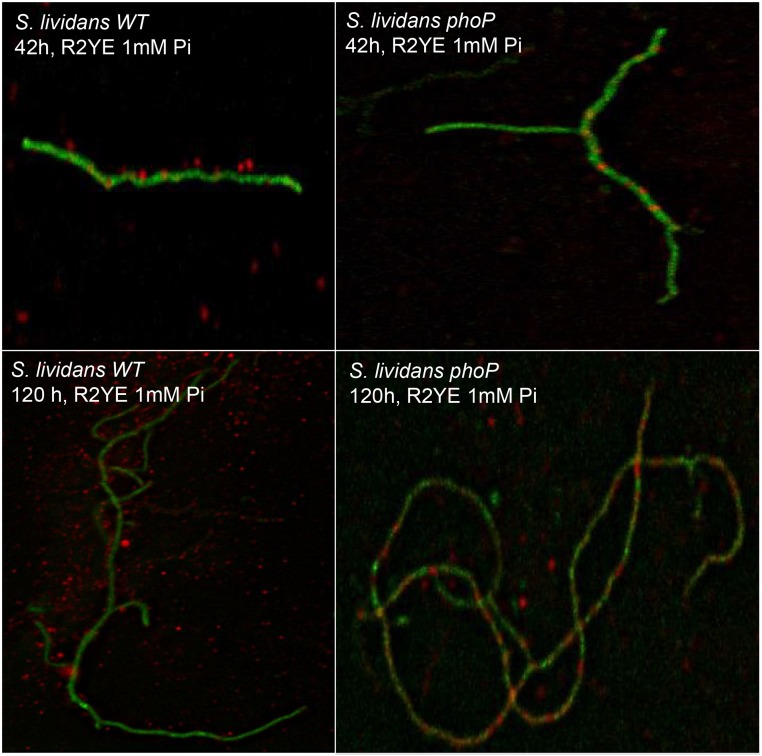
Fluorescent microscopic observations of mycelial fragments. Mycelial fragments of the WT and the *phoP* mutant of *S*. *lividans* TK24, grown for 42 h and 120 h on solid R2YE medium limited in Pi (1mM) were stained with DAPI and observed using fluorescent microscopy. The pictures provided are from a single image from the z series. The color chosen with Metamorph was green for DNA and red for polyP. The images indicate the presence of few red dots inside the mycelium of the WT strain and abundant red dots outside. Extracellular red dots were far more abundant at 120 h than at 42 h. In contrast, in the *phoP* mutant, at both times, most of red dots seen are inside the mycelium and very few outside.

**Fig 5:**
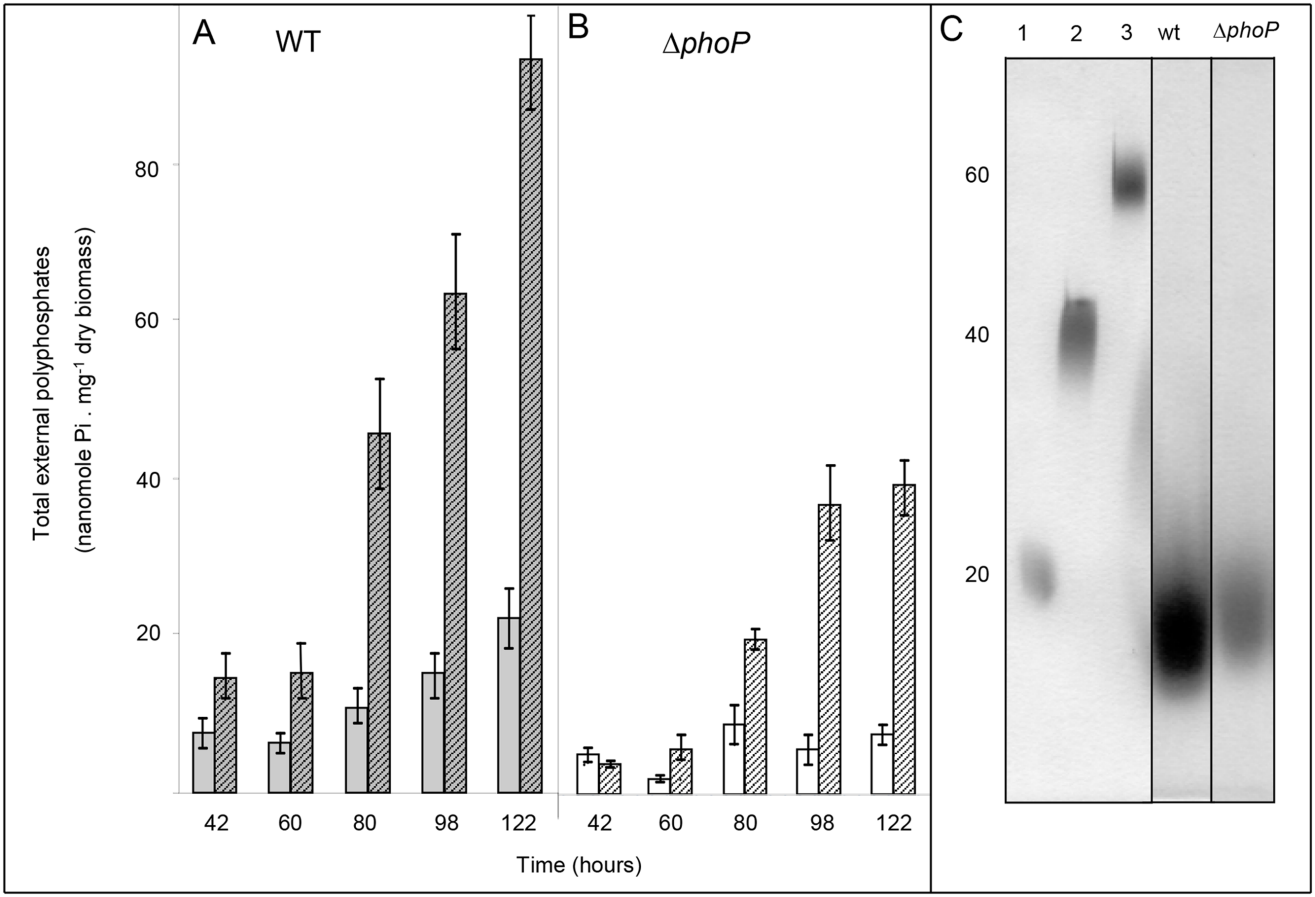
Extraction, quantification and size determination of extracellular polyP. 100mg of mycelium of the WT and *phoP* mutant, grown on a Pi limited solid R2YE medium, was washed with sterile water or with a solution of 5 mM EDTA. PolyP was extracted from the washing solutions, quantified and run on a on urea-polyacrylamide gel for size determination at different time points throughout growth. (A) The plain grey and striped grey histograms represent polyP found in the wash solution of the WT strain with water or with 5 mM EDTA, respectively. (B) The plain white and striped white histograms represent polyP found in the wash solution of the *phoP* mutant with water or with 5 mM EDTA, respectively. The error bars represent the standard deviation of three independent experiments. (C) Poly P collected after EDTA washing of 100mg of mycelium of the WT and *phoP* mutant strains after 80 h of cultivation was analyzed on a urea-polyacrylamide gel. Size standard polyP, approximate length 20, 40 and 60 residues, are shown in lanes 1, 2 and 3. The polyP is approximately 10 residues long in both strains and is more abundant in the WT than in the *phoP* mutant strain.

Fluorescent microscopy observations of mycelial fragments of *S*. *lividans* TK24 stained with DAPI suggested the presence of polyP, within but also outside the mycelial fragments (Fig 4). In order to verify and quantify this observation, the same amount of mycelium of the WT and the *phoP* mutant was washed either with water or with 5 mM EDTA. PolyP was extracted from these wash solutions and their Pi content was determined. Results in Fig 5A and 5B clearly indicated that some polyP is indeed present outside the mycelial fragments of both strains. More polyP was extracted when EDTA was present in the wash solution, indicating the existence of electrostatic interactions, involving divalent cations, between the cell wall of the bacteria and polyP. This study also revealed that polyP bound to the cell wall was approximately two fold more abundant in the WT strain than in the mutant (80 h). This indicated that the generation and subsequent externalization of short polyP is a process directly or indirectly dependent upon *phoP*. When this polyP was run on an acrylamide gel, the size range was similar in both strains and shown to be around 10 residues (Fig 5C).

## Conclusion

**Fig 6:**
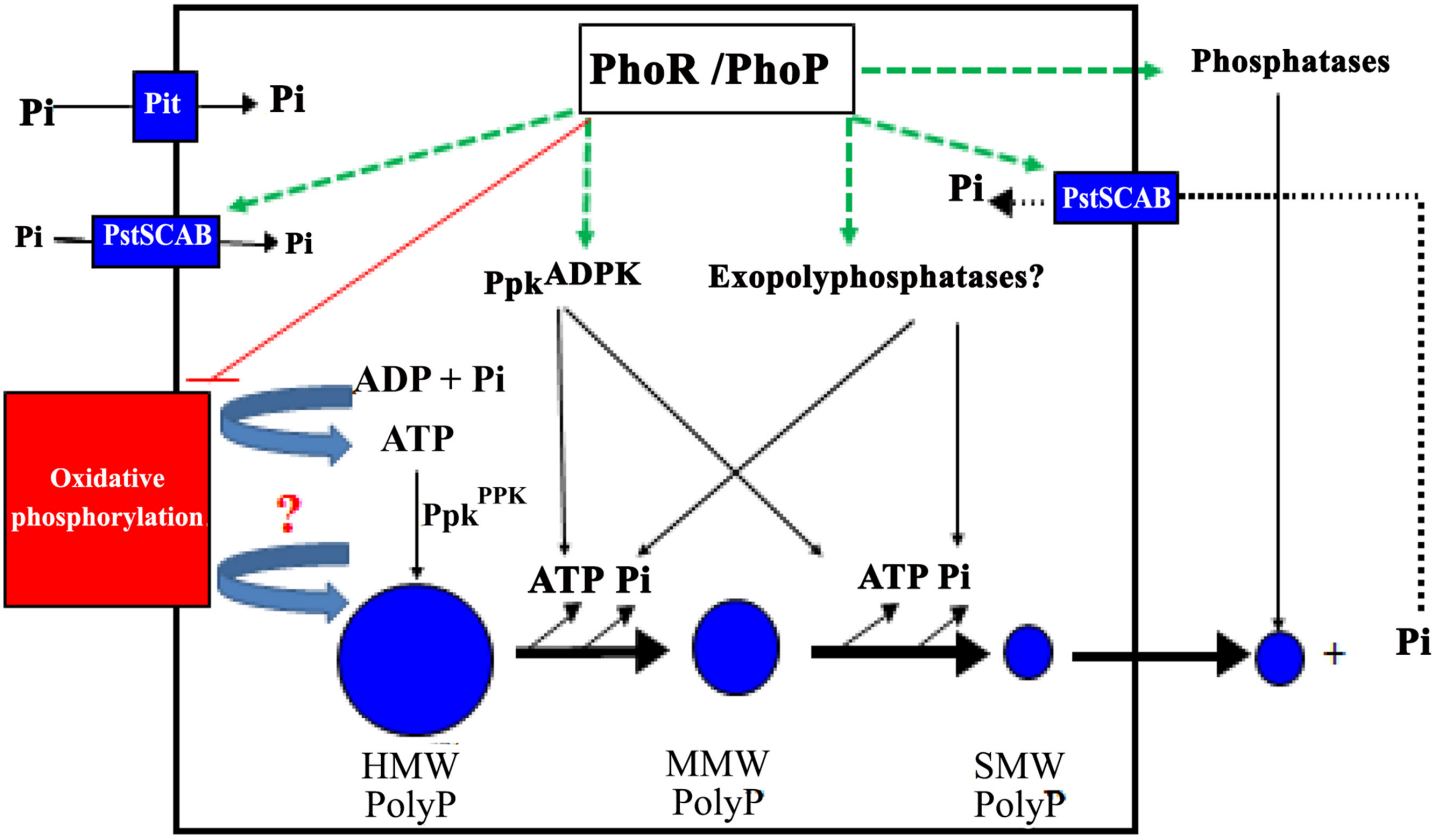
Schematic representation of the biosynthesis and fate of polyP in the WT and *phoP* mutant strains of *S*. *lividans* TK24. The various steps under the negative and positive control of the two component PhoR/PhoP system are shown in red and green, respectively. External Pi is taken up by specific (low and/or high affinity) Pi transport systems and is directly (by an unknown mechanism perhaps related to oxidative phosphorylation) or indirectly (*via* ATP biosynthesis and Ppk^PPK^) stored as polyP. PolyP abundance and chain length vary with the concentration of Pi in the growth medium. In conditions of Pi limitation, polyP would be degraded by various enzymes belonging to the *pho* regulon (exopolyphosphatases and Ppk^ADPK^). Short polyP, resulting from this degradation, is proposed to diffuse passively across the cell membrane. External polyP would then be degraded to Pi by secreted phosphatases belonging to the *pho* regulon and taken up by the high affinity transport system PstSCAB.

The picture that emerges from the present study is summarized in the schematic model shown in Fig 6. In *Streptomyces*, as in other bacteria, polyP content and polyP chain length are directly related to the Pi content of the growth medium and thus rely on an active Pi uptake system that, in *E*. *coli*, is regulated by *phoU* and *yjbB* [24]. The energetic vector and the molecular processes responsible for polyP synthesis remain to be identified. The dogma says that polyP is synthesized by PPK, an enzyme able to polymerize the gamma phosphate of ATP into polyP [25]. However, the fact that polyP remains abundant in *ppk* mutants of various bacteria including *E*. *coli* [26], *Pseudomonas* [27] and *Streptomyces* [28], suggests that at least one other major biosynthetic route remains to be discovered. Interestingly, in mammalian cells the inhibition of the ATP synthase and/or the disruption of the proton gradient, correlated with a lower polyP content [29]. Similarly, in the yeast *Saccharomyces cerevisiae*, inhibition of a vacuolar ATPase known to be involved in H^+^ gradient formation, was also correlated with a lower polyP content [30]. In both cases the authors proposed that the synthesis of polyP, as that of ATP, directly involves oxidative phosphorylation. However, one cannot exclude that in these cases the reduced ATP synthesis leads to an enhanced mobilization of polyP, resulting in a lower polyP content.

In our study we observed an unexpected higher content of ATP in the *phoP* mutant late in growth than in the WT strain in conditions of Pi proficiency. The major part of the ATP produced in the cell is produced by the ATP synthase coupled to the respiratory chain (oxidative phosphorylation) and interestingly Allenby *et al*. recently demonstrated that that some components of the respiratory chain were negatively regulated by PhoP [6]. We thus propose that this higher ATP content might, at least in part, be due to the de-repression of oxidative phosphorylation in the *phoP* mutant. High oxidative phosphorylation activity might also have a direct (as hypothesized in [29] and [30]) or indirect (via ATP synthesis and polymerization by Ppk) positive impact on HMW polyP synthesis. However, we rather believe that the greater abundance of HMW polyP in the mutant is due to the less efficient degradation of these polymers into shorter forms and Pi.

In Pi limitation, de-repression of oxidative phosphorylation is also likely to occur in the *phoP* mutant. However, this does not result in an ATP concentration higher than in the WT, maybe because the intracellular concentration of free Pi is too low to allow such synthesis. Our results also confirmed that, as expected, the overall polyP content was lower in Pi limitation than in Pi sufficiency, indicating that polyP synthesis was directly related to Pi content of the growth medium and to Pi uptake as found previously [24]. The degradation of long polyP into shorter forms and Pi was shown to be less efficient in the *phoP* mutant than in the WT, in both Pi conditions, accounting for the lower level of intracellular Pi in the mutant strain. This low free Pi, and thus ATP, levels could, at least in part, be responsible for the poor growth of this mutant in Pi limitation. PolyP is an important Pi and energy store and is probably degraded earlier in growth in Pi limitation than in Pi proficiency by enzymes belonging to the Pho regulon. These are exo-polyphosphatases that remain to be characterized in *S*. *coelicolor* (candidates genes are *sco3093*, *sco3348*, and *sco0757*) as well as Ppk, acting as an adenosine diphosphate kinase [17,31].

Finally and interestingly, our study also revealed that SMW polyP (10 residues long or less) were found tightly bound to the surface mycelium by electrostatic interactions involving divalent cations (most probably Mg^++^ and/or Ca^++^ ions that are abundant in R2YE). The presence of polyP attached to the cell surface was previously reported in other micro-organisms [32]. Two fold less SMW polyP was found attached to the surface of the mycelium of the *phoP* mutant compared to the WT simply because the mutant degrades less actively long polyP into shorter forms likely to diffuse across the cell membrane. An interesting possibility is that the degradation of extracellular polyP by secreted phosphatases, also belonging to the Pho regulon, might be responsible for the slight increase in the concentration of Pi observed at late time points (Fig 1A) providing free Pi for the whole population in conditions of Pi scarcity (Fig 6).
